# Triterpenoid derivatives inhibit Gli-mediated transcription in human glioblastoma cell line *via* direct interaction with Gli1

**DOI:** 10.1016/j.jbc.2025.110472

**Published:** 2025-07-10

**Authors:** Ivo Frydrych, Jiří Řehulka, Lucie Slavíková, Jiří Hodoň, Jan Pokorný, Veronika Šidová, Martina Medvedíková, Hanuš Slavík, Pavel Polishchuk, Tomáš Oždian, Jana Vrbková, Jan Šarek, Petr Džubák, Marián Hajdúch, Milan Urban

**Affiliations:** 1 Institute of Molecular and Translational Medicine, Faculty of Medicine and Dentistry, Palacky University and University Hospital Olomouc, Olomouc, Czech Republic; 2 Department of Organic Chemistry, Faculty of Science, Palacky University Olomouc, Olomouc, Czech Republic; 3 Institute of Molecular and Translational Medicine, Czech Advanced Technology and Research Institute, Palacky University Olomouc, Olomouc, Czech Republic

**Keywords:** triterpenes, betulinic acid, Hedgehog signaling pathway, transcription, glioblastoma, firefly luciferase, primary cilium, isothermal titration calorimetry (ITC)

## Abstract

The evolutionarily important Hedgehog (HH) signaling pathway plays a critical role in the development and progression of multiple solid tumors, such as basal cell carcinoma, medulloblastoma, rhabdomyosarcoma, and various gastrointestinal, pulmonary, and brain tumors. The proteins of the Gli (glioma-associated oncogene homologue) family are key mediators of the HH pathway. In the present study, we have focused on triterpenoid derivatives, which have been shown to induce apoptosis and inhibit HH signaling in rhabdomyosarcoma. Utilizing a U-87MG glioblastoma-derived reporter cell line, we screened a structurally diverse library of triterpenoid derivatives to identify potential antagonists of Gli-mediated transcription. We revealed two derivatives that not only selectively inhibited Gli-mediated gene transactivation but also displayed greater potency than the known Gli1 inhibitor GANT61. These compounds also demonstrated dose- and time-dependent inhibition of U-87MG tumor cell proliferation *in vitro*. Further mechanistic studies provided genetic evidence for the inhibition of the downstream HH pathway by these compounds, *via* reduced expression of Gli1 and its transcription targets. However, these compounds did not affect the ciliary localization of Smoothened (Smo). Our findings suggest that the observed inhibitory effects are likely due to a direct interaction between our compounds and Gli1.

Primary tumors of the central nervous system account for 12% of all cancers (1). Glioblastoma multiforme (GBM) is considered the most common and malignant primary brain tumor in adults. GBM represents ∼48% of all primary central nervous system tumors and ∼57% of all gliomas and are classified as Grade IV tumors by the World Health Organization (2). Although great progress has been made in the treatment of GBM in recent years, prognosis and survival are still unfavorable. The most common treatments for GBM include surgery, radiotherapy, chemotherapy, and targeted therapy (3, 4). Within chemotherapy, temozolomide represents the current gold standard (5). Treatment with temozolomide in combination with conventional radiation therapy has been found to dramatically extend the median survival of patients compared to those treated with radiotherapy alone. Despite that, the survival rate is still poor (6). In addition, since temozolomide is an alkylating drug, its benefit is limited to patients with low or absent activity of the DNA-repair enzyme O^6^-methylguanine DNA methyltransferase (7). Another limitation of temozolomide treatment is GBM resistance caused by deregulation of multiple signaling pathways. Therefore, alternative strategies involving the targeting of different cellular receptors, such as the vascular endothelial growth factor receptor, epidermal growth factor receptor, or platelet-derived growth factor receptor, have been considered for GBM treatment (8, 9). Other described targets are protein kinase C, mammalian target of rapamycin, or histone deacetylases (10). Some of the above-mentioned strategies have already been evaluated in clinical trials. Unfortunately, due to limited treatment options and heterogeneous patient responses to treatment, there is still a high demand for new therapeutic agents.

Recently, the Hedgehog (HH) signaling pathway has emerged as an attractive target for anticancer therapy due to its aberrant activation in a number of tumors, including glioblastoma (11, 12, 13, 14). Some studies have demonstrated the involvement of several developmental pathways (Wnt, Notch) in GBM progression, while HH signaling has an essential role in glioma stem-like cell proliferation and tumorigenesis (15, 16). Activation of canonical HH signaling occurs when one of the vertebrates’ possessed HH ligands, Sonic (Shh), Indian (Ihh), or Desert (Dhh), bind to the extracellular domain of the Patched (PTCH) transmembrane receptor. This binding relieves PTCH-mediated repression of the protooncogene Smo, which in turn transduces a signal to the final effectors of the HH pathway, Gli family (Gli1, Gli2, and Gli3) of zinc finger transcription factors, which translocate to the nucleus and regulate transcription of target genes. Within this family of transcription factors, Gli1 and Gli2 constitute key transcription effectors with regard to tumorigenesis, and constitutive activation of at least one of them is essential for cancer development (17). The importance of Gli factors for cancer development and progression is that they can activate the expression of a number of target genes involved in proliferation (*e.g.* Cyclin D1, N-Myc, and FoxA2), survival (Bcl-2), angiogenesis (Ang1/2), epithelial-mesenchymal transition (Snail 1, Sip1, Elk1) and stem cell self-renewal (Nanog, Sox2, Bmi1) (18, 19). Remarkably, Gli transcription targets also include Ptch1 and Gli1, which form a feedback loop of the HH pathway that enhances or represses the HH response. Other important members of the HH pathway acting between Smo and Gli are proteins exerting negative (Suppressor of Fused, Rab23, Ren) or positive (Tectonic, MIM/BEG4) effects on HH signaling (20, 21). Among all, Suppressor of Fused (SUFU) represents a key negative regulator of Gli proteins (22).

Currently, most medicinal chemistry efforts involving the HH pathway are focused on targeting Smo. Unfortunately, in some tumors, including medulloblastoma, glioma, pericytoma, prostate, and breast cancer, there are many alternative mechanisms for HH signaling activation through Smo downstream effectors, making Smo inhibitors ultimately ineffective. Well-documented examples of mechanisms leading to HH activation are mutation or overexpression of Gli1 (23), Gli2 (24), or SUFU (25), Gli1 chromosomal translocation (26), or Ren deletion (27). Another limitation of Smo antagonism is the occurrence of drug-resistance Smo mutations. Thus, inhibitors of Gli transcription, which is the terminal event in HH signaling, would have better applicability in HH-dependent tumors, regardless of the upstream pathway components responsible for activation. The Gli antagonists (GANTs) were discovered for the first time by Lauth *et al.* and named GANT61 and GANT58 (28). Both were reported to inhibit Gli-mediated gene activation, but GANT61 proved more potent inhibition on Gli1 and Gli2 in many cancer cell lines (29, 30, 31). Another Gli1 and Gli2 transcription inhibitor, arsenic trioxide, has been approved by the FDA for the treatment of acute promyelocytic leukemia (32). A complete review of Gli antagonists acting in direct or indirect ways is available elsewhere (33).

In 2010, Eichenmüller *et al.* described the inhibitory effect of betulinic acid triterpene on HH signaling in rhabdomyosarcoma (34). Motivated by this finding, we screened a library of structurally diverse triterpenoid derivatives as potential antagonists of GLI-mediated transcription, a key step in HH signaling. To address this goal, we developed and validated a cell-based assay using the U-87MG glioblastoma cell line. As a result, we identified two potent inhibitors of GLI-mediated transcription and subjected them to a detailed study of their mechanism of action.

## Results

### Chemistry

**Figure 1:**
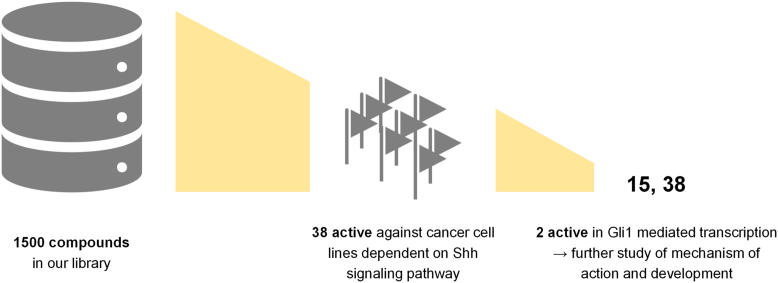
**Selection of compounds for this study**.

**Figure 2:**
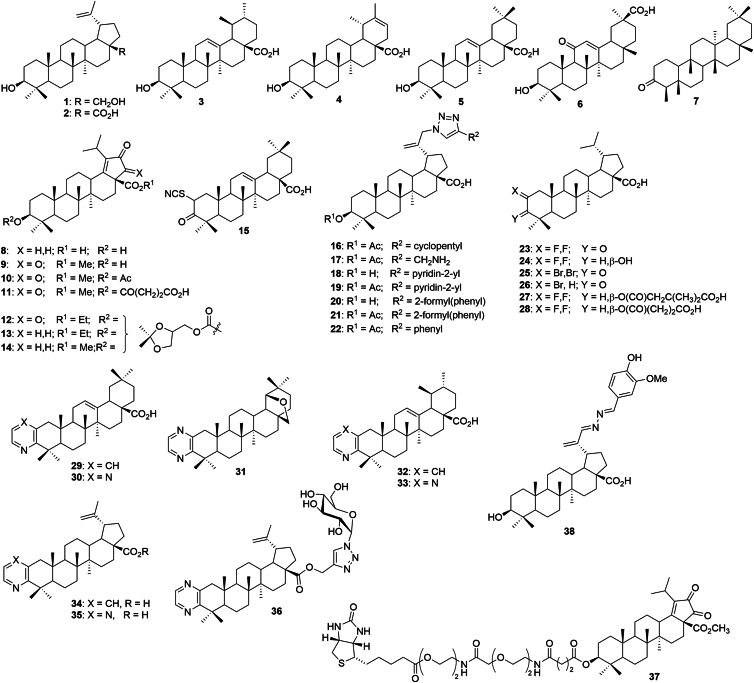
**Structures of all compounds included in this study**.

Based on the literature precedent which described the inhibitory effect of betulinic acid 2 on HH signaling in rhabdomyosarcoma (34), we did an extensive screening of our large library of triterpenoids (around 1500 compounds of a large structural diversity, Fig. 1). In this screening, we focused on the compounds that exhibited reasonable cytotoxicity in the HH signaling pathway dependent cancer cell lines (*e.g.*, U-87MG; T98G; DU-145). This screening yielded the first set of 38 hit compounds that were then tested for the activity in the HH signaling pathway (1–38, Fig. 2). Compounds 1 to 7 are common natural product triterpenoids that we routinely use as standards in biological assays (betulin 1, betulinic acid 2, ursolic acid 3, heterobetulinic acid 4, oleanolic acid 5, glycyrrhetinic acid 6, friedelin 7). Compounds 1 to 7 were purchased from the company Betulinines (www.betulinines.com) and used in assays and for the preparation of further derivatives 8 to 37. The synthesis and characterization of Compounds 8 to 37 have been published previously. The preparation of Compounds 8 and 9 is described in (35), Compounds 10 and 15 are in (36, 37), and Compounds 11 to 14 were patented in (patents WO2008037226A3; WO2001090046A1). Compounds 16 to 22 are described in (38), Compounds 23 to 28 in (39) and in (40), Compounds 29 to 36 in (41), Compound 37 in (42), and Compound 38 in (43).

### Biology

#### Effect of a small library of structurally diverse triterpenes on Gli1 activation and survival

In our initial studies, we focused on assessing the effect of a small triterpenes’ library on Gli-mediated transcription by using a cell-based reporter. We decided to target the Gli proteins because these transcription factors represent the indispensable and ultimate downstream effectors of the HH signaling pathway. We used U-87MG cells as an established *in vitro* model of glioblastoma that exhibits active HH signaling as indicated by Gli1 expression and nuclear localization. U-87MG cells were stably transduced with lentiviral particles expressing an inducible reporter construct to screen for Gli-mediated transcription (for a detailed description of reporter preparation, see chapter 2.2). The IC_50_ values for Gli-mediated transcription after 24 h treatment are summarized in Table 1. Within the set of 38 compounds investigated, half of them showed IC_50_ = 5 μM or lower. It is important to note that these IC_50_ correspond to experimental conditions comprising serum-reduced culture medium. These experimental conditions were selected because of the influence of growth factors on HH signalization and also because triterpenes have a high binding capacity to serum proteins, which is likely to lead to a significant reduction in biological activity. At the same time, we also measured the effect of the compounds on cell proliferation and survival by MTS assay to differentiate between specific Gli1 inhibition and nonspecific cellular responses. Based on the obtained IC_50_ values for survival (Table 1), which are much higher compared to the Gli1 IC_50_, it is obvious that Gli1 inhibition precedes proliferation inhibition or cell death induction. We subsequently performed the same screen in a standard culture medium containing 10% of FCS, which better reflects clinically relevant conditions. Not surprisingly, most of the compounds highly potent under serum-reduced conditions lost or at least significantly reduced their inhibitory effect on Gli-mediated transcription. The IC_50_ values for Gli1 inhibition in 10% serum medium increased 2–10-fold compared to serum-reduced medium (Table 1). A similar trend is evident for IC_50_ reflecting cell survival (Table 1). Nevertheless, two compounds, 15 and 38, retain low IC_50_ for Gli1 inhibition even under normal serum conditions (Table 1). Based on these results, we selected Compounds 15 and 38 for a detailed analysis of the mechanism of action. Chemically, 15 and 38 represent quite distinct classes of triterpenes, with 15 being a terpenoid E-*seco*-anhydride and 38 an azine modified with an aromatic substituent at the C-30 position. In our previous publications, we also tested the cytotoxicity of compounds 15 and 38 on a panel of tumor cell lines, including those derived from hematologic malignancies and solid tumors, some of which are known to be dependent on Hedgehog signaling (37, 43).

**Table 1:** Summary of IC_50_ (μM) corresponding to inhibition of Gli-mediated transcription and cell survival under normal and serum-reduced conditions after 24 h of compound treatment

Compound	Gli1_0.5% FCS	MTS_0.5% FCS	Gli1_10% FCS	MTS_10% FCS
1	4.2	25.2	34.0	˃50
2	46.5	˃50	˃50	˃50
3	3.9	24.9	33.5	˃50
4	4.4	˃50	28.0	˃50
5	8.6	32.4	˃50	˃50
6	28.4	˃50	˃50	˃50
7	˃50	˃50	˃50	˃50
8	20.0	˃50	33.4	˃50
9	7.4	˃50	48.0	˃50
10	5.0	41.4	32.1	˃50
11	17	33.4	37.9	˃50
12	˃50	˃50	˃50	˃50
13	10.3	˃50	31.2	˃50
14	˃50	˃50	˃50	˃50
15	3.9	6.1	17.5	45.3
16	6.3	44.8	32.1	˃50
17	12.1	28.6	29.0	˃50
18	˃50	˃50	43.2	˃50
19	4.9	29.8	˃50	˃50
20	6.3	48.5	27.1	˃50
21	3.8	48.9	˃50	˃50
22	3.9	˃50	28.0	˃50
23	3.9	34.1	˃50	˃50
24	3.8	16.8	23.9	˃50
25	3.1	7.3	26.9	˃50
26	4.5	26.9	34.2	˃50
27	3.6	16.3	31.1	˃50
28	4.1	32.6	˃50	˃50
29	4.2	40.5	33.8	˃50
30	4.9	34.9	39.1	˃50
31	˃50	˃50	˃50	˃50
32	4.3	31.6	37.5	˃50
33	4.3	34.5	47.1	˃50
34	27.8	˃50	˃50	˃50
35	3.7	22.3	˃50	˃50
36	8.1	37.8	24.9	˃50
37	˃50	˃50	˃50	˃50
38	6.0	25	17.0	48.9

Values represent the mean of three biological replicates. The standard deviation in cytotoxicity and reporter assays is typically within 10% of the mean value. Compounds with IC^50^ > 50 μM are considered inactive.

#### Effect of 15 and 38 on firefly luciferase inhibition

**Figure 3:**
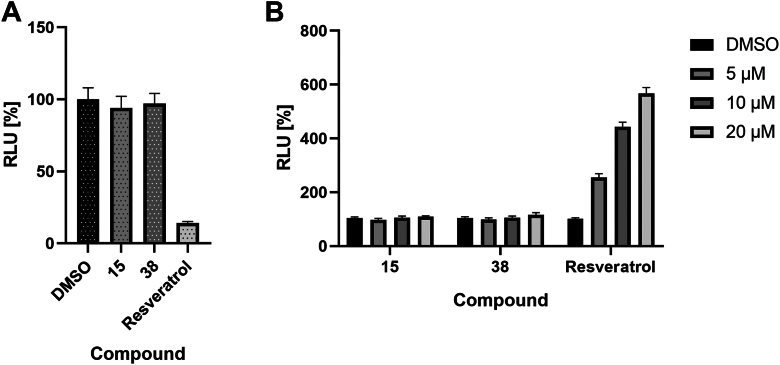
**Inhibitory effect of 15 and 38 on firefly luciferase.***A*, effect of the compound at 50 μM concentration on purified firefly luciferase. *B*, effect of the compound on firefly luciferase in cell-based assay. A concentration range of 5 to 20 μM was used. Resveratrol has been used as a positive control. Data represent the mean of three independent biological replicates performed in quadruplicates with standard deviations.

Since we used a cell-based assay with firefly luciferase as a reporter for screening, it was necessary to verify the possible inhibitory effect of active derivatives on luciferase itself, which can generate false-positive results. To exclude this possibility, we used two separate experiments. First, we incubated individual compounds with purified firefly luciferase (Fig. 3*A*) and in the second case, we used a cell model with constitutively expressed firefly luciferase (Fig. 3*B*; see section 2.6 for details regarding reporter development). According to the results, it is evident that at a concentration corresponding to the effective Gli-mediated transcription inhibition, there is no significant inhibitory effect of 15 or 38 on firefly luciferase in both the cell-based and recombinant luciferase assays. These findings indicate that the inhibitory effect of 15 and 38 is specific to Gli.

#### Time-dependent IC_50_ analysis for Gli-mediated transcription and survival

**Figure 4:**
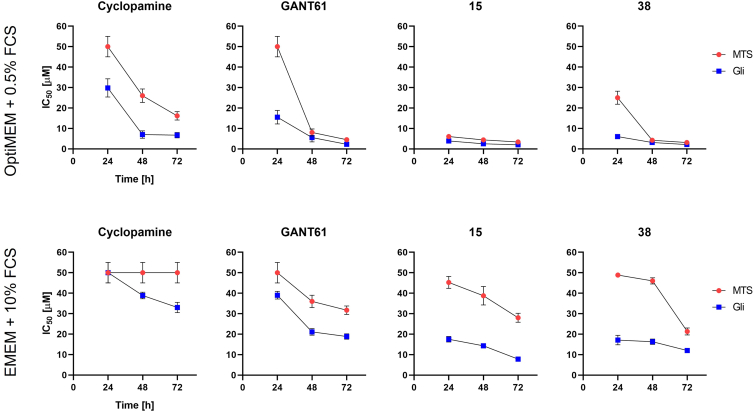
**Time dependent analysis of IC_50_ for Gli-mediated transcription and survival.***Blue points* represent IC_50_ corresponding to Gli, *red points* represent IC_50_ corresponding to cell survival. Data represent the mean of three independent biological replicates with standard deviations.

Following the results corresponding to the 24 h treatment, we further concentrated on the effect of 15 and 38 on Gli-mediated transcription at extended time points (48 and 72 h) to determine the improvement in inhibitory efficacy. For this study, we also included two well-known HH signaling pathway inhibitors, cyclopamine (Smo antagonist) and GANT61 (Gli agonist). The analysis was performed under two different culture conditions comprising normal (Fig. 4, lower part) and serum-reduced (Fig. 4, upper part) medium. Not surprisingly, we observed a significant decrease in IC_50_, proportional to the increase in incubation time. Following 72 h treatment, the calculated IC_50_ for Gli-mediated transcription by 15 and 38 at serum-reduced conditions correspond to 2.0 and 2.1 μM, respectively. Similarly, survival-related IC_50_ reached the lowest values at the 72 h time point, corresponding to 3.4 (15) and 3.1 (38) μM, respectively. As discussed in chapter 3.2.1, under normal serum conditions, significantly higher IC_50_ were found after 72 h of treatment, corresponding to 7.8 (15) and 12.0 (38) μM, respectively. At all measured time points, the IC_50_ values corresponding to survival are much higher than those corresponding to Gli-mediated transcription. This clearly indicates that inhibition of HH signaling unequivocally occurs prior to the induction of cell death. The results also showed that 15 and 38 exhibited a much stronger inhibitory effect on Gli-mediated transcription under both culture conditions compared to the known Gli1 inhibitor GANT61. Especially under normal serum conditions, the differences are very striking. On the other hand, the efficacy of the Gli1 upstream SMO antagonist Cyclopamine is very low, especially under normal serum conditions.

#### Effect of 15 and 38 on the mRNA and protein expression of Gli1 and Gli1 transcription targets

**Figure 5:**
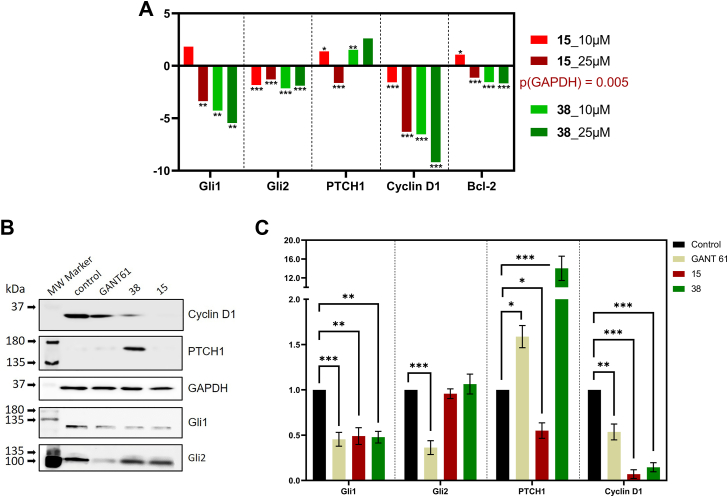
**Compounds 15 and 38 regulate key components of HH signaling.***A*, RT-qPCR analysis of genes related to the HH pathway in U-87MG reporter cells treated for 24 h with DMSO (Control) and 15 or 38 at a concentration of 10 or 25 μM. Relative quantification was used to evaluate the qPCR data. GAPDH was used to endogenously normalize Ct values for each marker. Fold changes were calculated from ΔΔCt values as averages of ΔCt values from treated samples *versus* ΔCt averages from controls. A multiple *t* test was used to examine statistical differences between treatment and control for each marker separately. *B*, Western blot analysis of HH pathway-related proteins in U-87MG reporter cells treated with DMSO (Control), GANT61, 15 or 38 at 25 μM for 24 h. A representative image from 3 replicates is shown. *C*, quantitative analysis of protein expression levels. The density of bands was analysed using ImageJ software (48). The relative protein intensity was calculated using GAPDH as a loading control and expressed as fold change from control (DMSO) set to 1. Multiple *t* test was used for data analysis in the same way as for qPCR. Means and standard deviations from three biological replicates were used to visualize the data in the presented bar graphs: ∗*p* < 0.05; ∗∗*p* < 0.01; ∗∗∗*p* < 0.001.

To examine the molecular mechanism of the phenotypic changes, we performed real-time quantitative PCR (RT-qPCR) and Western blot analysis to monitor the expression of critical components of the HH signaling pathway at both the mRNA and protein levels. We found that there are dramatic changes in the expression of Gli1 transcription targets. Importantly, Gli1 transcription targets include those involved in the feedback mechanisms of the HH (Gli1 and PTCH1). Thus, HH dependent upregulation of the Gli1 transcription factor is a positive feedback mechanism, whereas HH dependent upregulation of PTCH1 comprises a negative feedback mechanism of HH signaling. Despite the 15 and 38 structural differences, both molecules were able to interfere with the Gli1 targets mRNA expression level in a dose-dependent manner, as shown in Figure 5*A*. Both compounds significantly reduced the mRNA expression of Cyclin D1 and Bcl-2, which are important Gli1 targets involved in the regulation of cell proliferation and cell death processes, respectively. Different effects were observed on PTCH1 mRNA expression. While 15 at a higher concentration reduced PTCH1 mRNA level, the effect of 38 was the opposite for both concentrations used. Considering that PTCH1 HH dependent upregulation comprises a negative feedback mechanism, this effect induced by 38 could have a highly beneficial anti-tumor effect. Given the high degree of homology between Gli1 and Gli2, it is not surprising that both compounds also significantly reduced Gli2 mRNA expression. Protein expression analysis revealed a significant inhibitory effect of both compounds on Gli1 protein expression, which is in good agreement with the mRNA results. In contrast to Gli2 mRNA expression, no changes at the protein level were detected after 15 or 38 treatments. Similar to the effect on Cyclin D1 mRNA expression, a dramatic decrease in Cyclin D1 protein was observed, correlating well with the observed antiproliferative effect of 15 and 38 on tumor cells (Figs. 4 and 5, *B* and *C*). A nice correlation between the effect on mRNA and protein levels was also observed for PTCH1. Interestingly, 38 induced an extremely strong increase in PTCH1 expression, almost 16-fold compared to the untreated control. The Gli1 inhibitor GANT61 exhibited, except for Gli2, a comparable pattern of change in expression of the studied protein, similar to the derivative 38.

#### Effect of 15 and 38 on Smo ciliary localization

**Figure 6:**
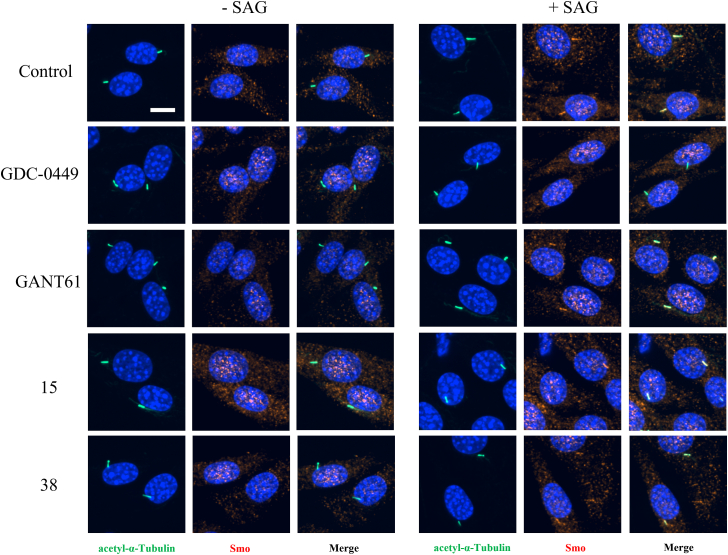
**Representative images of Smo localization in primary cilia of NIH 3T3 cells (co)treated with the HH agonist SAG, inhibitors, or derivatives 15 and 38.** The effect of SAG (5.0 nM), HH inhibitors GDC-0449 (5.0 μM), GANT61 (10.0 μM), and compounds 15 (2.5 μM), 38 (5.0 μM) or control (DMSO) was observed following 18 h incubation. Nuclei were visualized using Hoechst 33342 (*blue*), scale bar = 10 μm.

**Figure 7:**
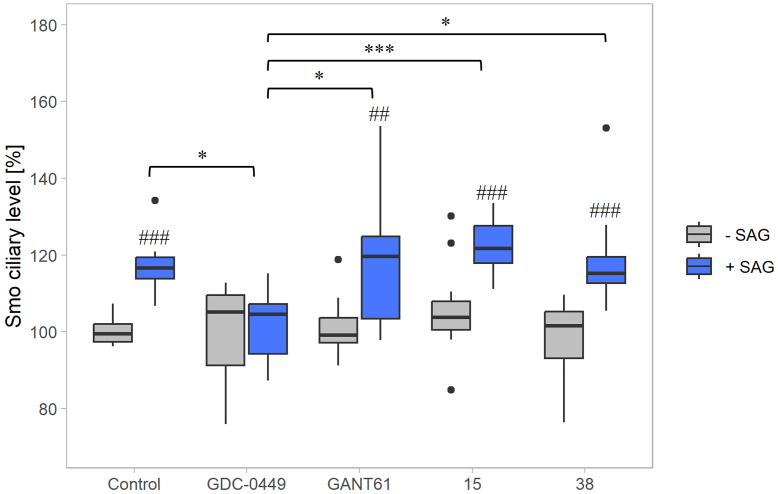
**Compounds 15 and 38 do not interfere with Smo localization upon HH activation.** The box plot shows median values obtained from three replicates performed in quadruplicates. NIH 3T3 cells were treated for 18 h with 5.0 nM SAG (*blue*) or without SAG (*grey*), HH inhibitors GDC-0449 (5.0 μM), GANT61 (10.0 μM), and compounds 15 (2.5 μM), 38 (5.0 μM), or control (DMSO). Statistical analysis of SAG treated and untreated samples was performed using 2-tailed two-sample *t* test or Wilcoxon test, respectively: #*p* < 0.05; ##*p* < 0.01; ###*p* < 0.001. The Kruskal-Wallis test with Dunn´s *post hoc* tests (Bonferroni correction applied) was used to compare SAG and compound co-treatment: ∗*p* < 0.05; ∗∗*p* < 0.01; ∗∗∗*p* < 0.001.

To further assess the effect of the tested compounds on the HH pathway, we monitored the presence of Smo in the primary cilium of the NIH 3T3 mouse cell line upon treatment with the studied compounds. In response to HH activation, Smo translocates into the primary cilium (44, 45). The accumulation of Smo in cilia was monitored using a specific antibody and colocalization with acetyl-α-tubulin which served as a cilia marker. Incubation was performed with and without simultaneous HH pathway activation by 5 nM Smo agonist (SAG). To avoid any cytotoxic effect of the compounds, a low 5 nM concentration of SAG was used to induce Smo translocation into cilia. The Smo inhibitor GDC-0449 and the Gli1 antagonist GANT61 were used as inhibitors of HH signaling with a different mode of action. Gli1 antagonists such as GANT61 do not modulate the level of Smo in cilia, in contrast to GDC-0449, which acts upstream of Gli and prevents Smo translocation into cilia. Positive controls and Compounds 15 and 38 itself did not alter Smo localization in SAG untreated cells (Fig. 6). SAG induced a statistically significant increase in the intensity of Smo in cilia in control cells and cells simultaneously treated with GANT61, 15, or 38 (Figs. 6 and 7). On the contrary, GDC-0449 completely prevented Smo accumulation in the cilia of SAG-activated cells (Figs. 6 and 7). The results indicate that Compounds 15 and 38 do not act as Smo inhibitors in the same way as GDC-0449. Our results demonstrate that derivatives 15 and 38 share a similar pattern to GANT61 and support the hypothesis that they modulate HH signaling at the Gli1 level.

#### ITC study of the interaction of 15 and 38 with recombinant Gli1 protein

Taking all the results of the biochemical analyses together, we assumed that our compounds might have a downstream Smo effect within endogenous HH signaling. Since 15 and 38 proved similar biological effects like GANT61 across parameters measured, we raised a question regarding the possible interaction of 15 or 38 with Gli1. Therefore, we used the isothermal titration calorimetry (ITC) method to show whether these compounds bind to Gli1. In these experiments, we used a recombinant protein corresponding to the zinc finger domain of human Gli1 (amino acids 222–400) with a C-terminal His tag, as detailed in section 4.1 of the Experimental Procedures. Additional confirmation of its purity, molecular weight, and C-terminal His tag of the recombinant protein was obtained by gel electrophoresis and mass spectrometry, as shown in Figs. S1 and S3.

**Figure 8:**
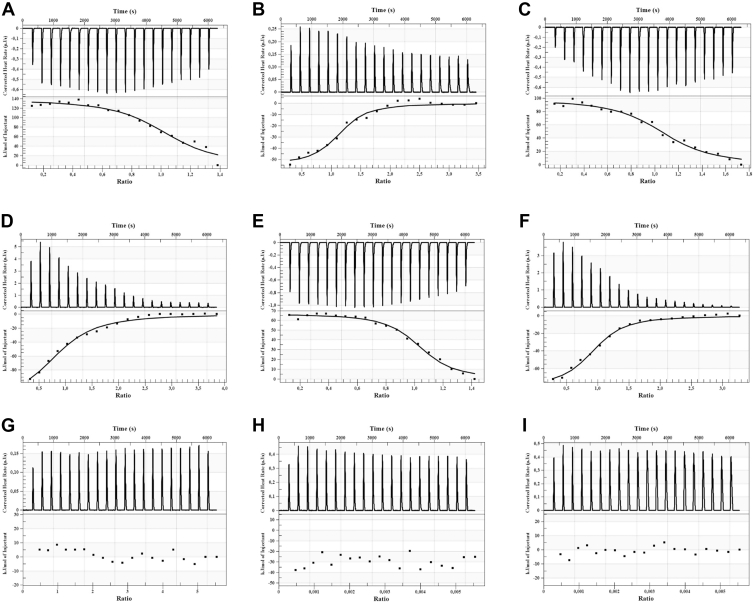
**ITC analysis of GANT61, 15, 38, and cyclopamine binding to Gli1.** Representative ITC data showing binding of GANT61, Cyclopamine, 15, and 38 to purified Gli1 (*A–G*) and interaction of 15, and 38 with Triton X-100 itself (*H* and *I*). *A*, GANT61. *B*, GANT61 in the presence of Triton X-100. *C*, compound 15. *D*, compound 15 in the presence of Triton X-100. *E*, compound 38. *F*, compound 38 in the presence of Triton X-100. *G*, cyclopamine. *H*, interaction of 15 with Triton X-100. *I*, interaction of 38 with Triton X-100. (*Top*) Raw data plot of heat flow *versus* time for titration of Gli1 with GANT61 (15, 38, cyclopamine) or Triton X-100 titration with 15, and 38. (*Bottom*) The plot of molar enthalpy change *versus* the molar ratio of GANT61 (15, 38, cyclopamine)/Gli1 or 15 (38)/Triton X-100 M ratio.

ITC measures the heat released or absorbed during the interaction and provides thermodynamic information with relevance to the binding affinity of the small molecule to the protein. The thermodynamic parameters for the interaction of the tested compounds with Gli1 obtained from ITC are listed in Table 2. The measured enthalpies for the interactions of GANT61 and derivatives 15 and 38 with Gli1 protein and *Ka* values indicate moderate affinity (Table 2). The *Ka* value for the interaction with 38 was comparable to that for GANT61, although the interaction with 15 appears to be somewhat weaker. The stoichiometric binding number (n) for all compounds at all experimental settings is approximately 1, indicating a 1:1 interaction between the compound and Gli1 molecules (Table 2). Negative *ΔG* values for all interactions studied indicate their spontaneous nature (Table 2). Representative examples of calorimetric titration profiles showing compound−protein interaction are shown in Figure 8. The results also showed positive entropy level change for interaction with all compounds (Table 2), indicating the presence of hydrophobic interactions on the protein surface. Therefore, we performed another set of experiments in the presence of 1 mM Triton X-100, which is known to act as an inhibitor of hydrophobic interactions (Fig. 8, *B*, *D* and *F*). As a negative control, we used the Smo antagonist Cyclopamine, which exhibited no ITC interaction with the Gli1 protein (Fig. 8*G*).

**Table 2:** Thermodynamic and binding parameters defining the interactions of GANT61, 15, and 38 with Gli1 based on ITC titrations

Gli1	Compd no.	*K*_a_	Δ*H*	*n*	*K*_d_	Δ*S*	Δ*G*
		(1/M)	(kJ mol^−1^)		(M)	(J mol^−1^ K^−1^)	(kJ mol^−1^)
25 mM HEPES, pH 7.4	GANT61	8.72 × 10^6^	137.50	1.035	1.14 × 10^−7^	594.1	−39.6
	15	2.59 × 10^6^	97.48	1.079	3.8 × 10^−7^	449.7	−36.6
	38	7.78 × 10^6^	66.47	1.018	1.28 × 10^−7^	354.9	−239.3
25 mM HEPES, pH 7.4 + 1 mM Triton X-100	GANT61	2.03 × 10^6^	−54.12	1.118	0.49 × 10^−7^	−41.59	−41.7
	15	1.42 × 10^5^	−125.9	0.903	7.05 × 10^−6^	−327.3	−28.3
	38	4.03 × 10^5^	−80.13	0.931	2.48 × 10^−6^	−161.5	−31.9

ΔG, Gibbs energy change; ΔH, enthalpy change; ΔS, entropy change; K_a_, association constant; K_d_, dissociation constant; n, stoichiometry.

## Discussion

The current study evaluated the effect of various triterpenoid derivatives on Hedgehog (HH) pathway, a pathway that is critically involved in the development and progression of several cancers. Building on existing literature, pentacyclic triterpene betulinic acid 2 has previously been reported to inhibit Sonic Hedgehog signaling in the HaCaT reporter cell line and has demonstrated efficacy in *in vivo* models like RMS-13 (alveolar rhabdomyosarcoma) xenograft (34, 46).

From our library of triterpenoid compounds, which includes both semi-synthetic derivatives and naturally occurring structures, we identified two novel antagonists of the Sonic Hedgehog pathway: Compounds 15 and 38. To ensure the reliability of our findings, we took special precautions to avoid common pitfalls in drug screening. To exclude luciferase inhibition artifacts, we verified that the tested compounds did not interfere with the reporter enzyme (47).

Our cell-based experiments were conducted under two distinct conditions: medium supplemented with 10% FCS and reduced FCS. This approach was chosen to account for the influence of growth factors present in serum on HH signaling. In line with this, we observed variations that were indeed dependent on the culture conditions. This highlights an important pharmacological consideration as betulinic acid has been reported to bind to human serum albumin (48), which could significantly affect pharmacokinetic properties (49).

In our previous studies, Compounds 15 and 38 also demonstrated cytotoxic effects in a range of tumor cell lines, including both HH-dependent and HH-independent models. This broader anticancer activity suggests that their mechanism of action may not be limited solely to Gli1 inhibition. Compound 38 in particular exhibited higher potency, implying additional interactions with cellular targets beyond the HH pathway. Such multi-target potential is of interest for the development of anticancer agents, especially in the context of tumor heterogeneity and resistance mechanisms. Nevertheless, further studies will be needed to elucidate the full spectrum of molecular interactions and confirm whether these compounds modulate parallel signaling pathways involved in tumor cell survival.

To further clarify their mechanism, we investigated the molecular targets of compounds 15 and 38 within the HH pathway. Given the well-documented importance of Gli1 in tumor development and progression (50, 51, 52) we sought to determine whether these compounds directly targeted Gli1 or act upstream, *e.g.*, at the level of Smoothened (Smo). Automated confocal microscopy revealed that neither compound inhibited Smo translocation into the primary cilium, suggesting a mechanism of action downstream of Smo, consistent with the profile of known Gli1 inhibitors such as GANT61. This hypothesis was supported by isothermal titration calorimetry (ITC) data, which demonstrated direct binding of both compounds to Gli1. To ensure the reliability of these findings, we verified the identity, purity, and molecular weight of the recombinant Gli1 protein fragment (amino acids 222–400) by SDS-PAGE, which confirmed consistency with the expected band pattern. To validate that the recombinant Gli1 fragment used in ITC assays is biologically active and capable of specific DNA binding, we performed an electrophoretic mobility shift assay (EMSA) using a 27-mer duplex DNA oligonucleotide containing a validated Gli-binding site (see Fig. S5). As shown, the recombinant Gli1 protein produced a concentration-dependent mobility shift of the DNA duplex, confirming its ability to form DNA–protein complexes *in vitro*. This confirms that the selected fragment retains its functional DNA-binding properties while likely benefiting from improved solubility and structural stability compared to full-length Gli1, which contains extensive intrinsically disordered regions.

As triterpenoids are known to exhibit high binding affinity to serum proteins, which can complicate the interpretation of specificity in biophysical assays, we included cyclopamine, a structurally related SMO-targeting terpene as a negative control. Cyclopamine did not exhibit measurable binding to Gli1, supporting the specificity of interaction observed for compounds 15 and 38. While additional controls such as comparisons to unrelated zinc finger proteins or site-directed mutagenesis could further strengthen these conclusions, their applicability is limited by the conformational flexibility and only partial structural resolution of Gli1.

Interestingly, the binding of both GANT61 and Compounds 15 and 38 to Gli1 was found to be endothermic, a less common mode of interaction. Similar thermodynamic profiles have been reported for the binding of Indinavir to the HIV-1 protease (53), BP-1-102 to STAT3 (54), and positively charged molecules with glycosaminoglycans (55). The positive entropy changes observed in our data (Table 2) indicate the involvement of hydrophobic interactions on the protein surface. To test this, we repeated ITC experiments in the presence of 1 mM Triton X-100, a known disruptor of hydrophobic interactions (56). Under these conditions, we observed a shift in thermodynamic profiles from endothermic to exothermic, with a corresponding change in enthalpy (Fig. 7, *B*, *D* and *F*; Table 2). Additional control experiments showed no interaction of the tested compounds with Triton X-100 micelles alone (Fig. 7, *H* and *I*), suggesting that the observed heat release indeed results from interaction with Gli1.

To determine whether the interaction of Compounds 15 and 38 with Gli1 functionally impairs its DNA-binding ability, we performed EMSA using the same recombinant protein fragment (residues 222–400) and a validated 27-mer GLI-binding site oligonucleotide. Despite the functional similarity of these compounds to GANT61—including inhibition of Gli-dependent transcription—neither compound altered DNA–protein complex formation at concentrations up to 50 μM (Fig. S6). While these results may appear inconsistent with the thermodynamically confirmed interaction between compounds 15 and 38 and Gli1 observed by ITC, they more likely reflect a distinct mechanism of action. The compounds may not directly interfere with the DNA-binding interface but rather bind to allosteric or adjacent regulatory sites that modulate Gli1 activity in a more complex manner, without disrupting *in vitro* DNA recognition; however, they are capable of impairing transcriptional function in the cellular context. Further mechanistic studies will be required to clarify this apparent discrepancy.

To gain additional insight into the mechanism of Gli1 inhibition, we performed molecular docking of Compounds 15 and 38 to the zinc finger domain of Gli1 (PDB ID: 2GLI). The docking suggested that in the presence of DNA, both compounds tend to bind within the DNA-binding groove, while in its absence, they interact with shallow pockets on the protein surface near the DNA-binding site. These observations were followed by 50 ns molecular dynamics simulations of top docking poses, which showed substantial conformational changes in the protein, likely reflecting its intrinsic flexibility in the absence of DNA. Although these data indicate that Compounds 15 and 38 may interfere with Gli1-DNA interaction, the limited structural information available for Gli1 (only ∼25% of its sequence is resolved) restricts definitive conclusions. The unresolved regions, which may stabilize the DNA-binding domain or mediate protein–protein interactions, remain inaccessible to accurate modeling and could be critical for understanding ligand binding and functional inhibition.

To complement these structural predictions with functional evidence, we analyzed Gli1 protein levels by Western blotting, which revealed a concentration-dependent downregulation following treatment with Compounds 15 and 38. This effect was subsequently confirmed at the mRNA level by quantitative PCR, suggesting that the observed suppression of Gli1 activity may involve transcriptional or post-transcriptional regulatory mechanisms. The observed downregulation of Gli1 protein levels may result from multiple mechanisms. In addition to direct interference with its DNA-binding function, the compounds may promote Gli1 degradation *via* enhanced ubiquitination or other post-translational modifications, or by modulating its interaction with regulatory proteins such as SuFu. These combined effects could contribute to the observed loss of Gli1 activity and suggest that 15 and 38 act as multifaceted inhibitors of this oncogenic transcription factor.

Future work will focus on identifying the precise binding sites of these derivatives on Gli1 and evaluating their effects on key mechanisms of tumor cell proliferation, particularly in cancers with aberrant HH signaling, such as medulloblastoma or rhabdomyosarcoma. Although the compounds described in this study were not developed as clinical drug candidates at this stage, their biological activity and specificity toward Gli1 provide a valuable foundation for further development. The next logical steps would involve conducting *in vivo* studies to evaluate the bioavailability and therapeutic efficacy in animal models of HH pathway-dependent cancers. However, given the early-stage nature of these compounds and the exploratory character of our screening approach, we consider *in vivo* studies to be premature at this point. Instead, our work provides a comprehensive basis for prioritizing the most promising derivatives for further optimization and preclinical validation. We also plan to evaluate ADME (Absorption, Distribution, Metabolism, and Excretion) properties of these derivatives, particularly given the high lipophilicity commonly associated with triterpenoid scaffolds, which will be essential for understanding their pharmacokinetic behavior and therapeutic potential.

Overall, our results establish a strong foundation for the optimization of these compounds and the further development of targeted therapies against HH pathway-driven tumors.

## Experimental procedures

### Cell lines, chemicals, and antibodies

Human glioblastoma U-87MG cells (HTB-14), human lung carcinoma A-549 cells (CCL-185), and mouse embryonic fibroblast NIH 3T3 cells (CRL-1658) were purchased from the American Type Culture Collection (ATCC) and cultured in DMEM medium (Sigma-Aldrich) supplemented with 10% of FCS (Sigma-Aldrich) in a CO_2_ incubator at 37 °C. For the cell-based reporter assay, U-87MG cells were transferred to serum reduced OptiMEM medium (Invitrogen) supplemented with 0.5% of FCS, 1 mM of sodium pyruvate (HyClone) and 1× concentrated non-essential amino acid solution (HyClone). GANT61 (sc-202630A) and Smoothened agonist (SAG; sc-202814) were obtained from Santa Cruz. Cyclopamine (BPS-27013) and GDC0449 (BPS-27010) were obtained from BPS Bioscience. All inhibitors and agonists were dissolved in DMSO (Sigma-Aldrich) to obtain a 10 mM stock solution. Primary antibodies against Gli1 (ab134906), Gli2 (ab26056), Cyclin D1 (ab134175), PTCH1 (ab53715) and GAPDH (ab9485) were purchased from Abcam and all were used at 1:1000 dilution. Specificity of antibodies was validated by a vendor. Secondary antibodies produced in mouse or rabbit and conjugated with horseradish peroxidase (HRP) were purchased from Sigma-Aldrich and used at a 1:10,000 dilution. Recombinant Gli1 protein (MBS2889276) was purchased from MyBioSource. According to the manufacturer’s datasheet, the recombinant protein corresponds to a partial His-tagged fragment of human Gli1 (UniProt P08151), spanning amino acids 222 to 400, expressed in *E. coli*. The purity and sequence of the recombinant protein were verified using gel electrophoresis and mass spectrometry (see Supporting information). Phenazine methosulfate (PMS) and 3-(4,5-dimethylthiazol-2-yl)-5-(3-carboxymethoxyphenyl)-2(4-sulfonyl)-2Htetrazolium (MTS) were purchased from Sigma-Aldrich. Britelite plus reagent was obtained from PerkinElmer.

### U-87MG0derived Gli reporter development

The U-87MG cells were stably transduced with commercial lentiviral particles (cignal Lenti Gli Reporter Assays; Qiagen GmbH; CLS-3030L) carrying a Gli1 responsive element coupled to the firefly luciferase reporter gene. Monoclonal cell lines were generated from the polyclonal population by single cell sorting. At least 50 clones were validated using GANT61 and the best responding clone was used for further screening of the triterpene library.

### Cytotoxicity assay

The MTS cytotoxicity assay was performed as described in our previously published work (37). Briefly, U-87MG Gli1 reporter cells were seeded into 384-well transparent microplates at a density of 1.8 × 10^3^ cells per well. After 24 h, all test compounds were added to the microplates at a concentration range of 50 to 0.1 μM using an ECHO 550 (Labcyte) acoustic liquid handler. DMSO was added to the control wells in a volume corresponding to the maximum treatment concentration. Treated cells were incubated at 37 °C with 5% CO_2_ atmosphere for 24 h and then MTS/PMS solution (5 μl) was dispensed into the microplates using a Multidrop Combi dispenser (Thermo Fisher Scientific). The microplates were further incubated for 2 to 3 h until visible formazan crystals formed, and absorbance at 490 nm was measured using an EnSpire plate reader (PerkinElmer). After the subtraction of the blank, IC_50_ values were calculated using Dotmatics software (version 5.5; Dotmatics, Ltd). The data used for analysis were the results of three independent experiments.

### Gli reporter assay and time dependent analysis of IC_50_ for Gli

The Gli reporter cell line was seeded into white opaque 384-well microplates at a density of 2.5 × 10^3^ cells per well. After 24 h incubation at 37 °C in a 5% CO_2_ incubator, DMEM medium containing 10% of FCS was replaced with OptiMEM medium containing 0.5% of FCS in half of the plates. All analyzed compounds, including positive controls, were added to the microplates at final concentrations ranging from 0.1 to 50 μM using an ECHO 550 acoustic liquid handler and treated cells were incubated for 24, 48 and 72 h. At the end of the treatment period, the Britelite plus reagent was added and the luminescence signal proportional to Gli1 activation was measured using an EnSpire plate reader. After the subtraction of the blank, the inhibitory effect of the compounds was calculated as the percentage of luminescence of control (DMSO) set to 100%. IC_50_ values were calculated using Dotmatics software (version 5.5; Dotmatics, Ltd). Data from three biological replicates were used for analysis.

### Recombinant firefly luciferase assay

Recombinant firefly luciferase (SRE0045; Sigma Aldrich) was dissolved in 0.1% BSA/PBS to a final concentration of 0.01 μg/ml. Aliquots of 25 μl luciferase solution were dispensed into a white opaque 384-well microplate and then 25 μl of the analyzed compounds were added to the microplate at a final concentration of 0.1 to 50 μM. The plates were incubated for 30 min at RT in the dark. Immediately after adding 50 μl of Britelite plus reagent, the luminescence signal proportional to firefly luciferase activity was measured using an EnSpire plate reader. After subtracting the blank, the effect of the compound was calculated as the percentage of luminescence of the control (DMSO) set to 100%. The data used for the analysis were from three independent experiments.

### Luciferase cell-based assay and its development

A-549 cells were stably transduced with commercial lentiviral particles (Cignal Lenti positive control; Qiagen GmbH; CLS-PCL) constitutively expressing the firefly luciferase reporter gene. Monoclonal cell lines were generated from the polyclonal population by single cell sorting. At least 30 clones were validated using resveratrol, a potent firefly luciferase inhibitor. The best responding clone was used for further screening of the triterpene library. Briefly, the reporter clonal cell line A-549 was seeded into white opaque 384-well microplates at a density of 2.8 × 10^3^ cells per well. Following a 24 h incubation at 37 °C with 5% CO_2_ and 100% humidity, analyzed compounds were added to the microplates at final concentrations ranging from 0.1 to 20 μM using an ECHO 550 acoustic liquid handler and treated cells were incubated for an additional 24 h. Immediately after the addition of the Britelite plus reagent, a luminescence signal proportional to firefly luciferase transcriptional activity was measured using an EnSpire plate reader. After subtraction of the average background luminescence (control wells without cells), the effect of the compound was calculated as the percentage of luminescence of the control (DMSO) set to 100%. The data used for the analysis were from three independent experiments.

### RT-qPCR setup

U-87MG cells were washed twice with PBS solution by resuspension and centrifugation at 1000*g*/3 min. The final pellet was lysed in 1 ml TRI Reagent (Molecular Research Centre) and stored at −20 °C until RNA purification. Total RNA was extracted according to the manufacturer’s instructions. RNA concentration and purity were assessed using a Nanodrop ND 1000 instrument (ThermoScientific). For reverse transcription, samples were denatured at 3 μg RNA with 0.3 μg of Random Primers (Promega) at 70 °C for 5 min in a 19.5 μl volume of DEPC treated water (Ambion). Samples were placed on ice immediately after incubation. Then 6 μl of RevertAid 5× RT buffer (Fermentas), 3 μl of 10 mM deoxyribonucleotide triphosphates (dNTPs), and 0.75 μl of 40 U/μl RNAsin ribonuclease inhibitor (Promega) were added. Samples were incubated for 5 min at room temperature. In the last step, 150 U of RevertAid Moloney Murine Leukemia Virus reverse transcriptase (Fermentas) was added to each tube and the samples were incubated for 10 min at room temperature. Finally, samples were incubated at 42 °C for 60 min and then at 70 °C for 10 min. The prepared cDNA was stored at −20 °C until qPCR. PCR reactions were performed on a LightCycler 480 II/96 (Roche) in triplicates of 20 μl using the following temperature programs: for Gli1, PTCH1 and Cyclin D1 15 min at 95 °C and 45 cycles of 15 s at 95 °C, 10 s at 62 °C, and 15 s at 72 °C, followed by a final 10 min incubation at 72 °C; for Gli2 and Bcl2 15 min at 95 °C and 45 cycles of 15 s at 95 °C, 15 s at 60 °C, and 15 s at 72 °C, followed by a final 10 min incubation at 72 °C. Mixture composition and primer designs were adapted from previous studies (57, 58).

### Western blot

Western blot analysis was performed according to the protocol described elsewhere (59). Detailed information on the antibodies used and their dilution is given in Section 2.1.

### Cilia formation and Smo ciliary localization in NIH 3T3 cells

NIH 3T3 cells were seeded into 384-well Cell Carrier-Ultra plates (PerkinElmer) at a density of 1 × 10^4^ cells per well in 25 μl DMEM medium. After 6 h, the full growth medium was aspirated and replaced with DMEM containing 0.5% of FCS. Following a 24 h incubation that allows cilia formation, cells were treated with compounds using an ECHO 550 and incubated for additional 18 h. Then the cells were washed in PBS and fixed with 4% formaldehyde solution (Sigma-Aldrich) for 20 min.

### Immunofluorescence and microscopy

Fixed cells were permeabilized by 0.25% Triton X-100 (Sigma-Aldrich) and nuclei were stained with 10 μM Hoechst 33342 (Thermo Fisher Scientific) for 30 min. Proteins of interest were visualized using primary antibodies against Smo (sc-166685; Santa Cruz), acetyl-α-Tubulin (Lys40) (#5335; Cell Signaling Technology) and secondary antibodies conjugated with Alexa Fluor 488 and 568 (Thermo Fisher Scientific). Specificity of primary antibodies was validated by a vendor. Each well was acquired using an automated microscopic platform (Yokogawa CV8000, 60 × water immersion objective), with 6 microscopic fields per well, quadruplicates in three biological replicates. Images were analysed and Smo intensity was quantified using Columbus image analysis software (PerkinElmer).

### Isothermal titration calorimetry

The interactions of the compounds with Gli1 protein were studied by isothermal titration calorimetry (ITC). ITC experiments were performed at 25 °C using a Nano ITC Low Volume (TA Instruments). During all measurements, a total of 20 injections of 16 μM of the studied compound (2.5 μl each) were titrated into 250 μl of protein (1 μM) at 300 s time intervals, with a stirring speed of 250 rpm. All ITC experiments were conducted with degassed buffered solutions of 25 mM HEPES buffer, pH 7.4, in the presence of 1% DMSO. Control experiments were comprised of the titration of each complex solution into the buffer. Corrected data refer to the experimental data after subtracting the control data from the titration of the compounds into the buffer. The resulting thermograms were analyzed using the “Independent” model in NanoAnalyze software (TA Instruments). To determine the nature of drug-protein interactions, we performed additional titration experiments in the presence of 1 mM Triton X-100 to inhibit hydrophobic interactions.

## Patents

Patents WO2008037226A3; WO2001090046A1, patent application CZ 2022-277.

## Data availability

All data presented in this paper are contained within the manuscript.

## Supporting information

This article contains supporting information (60).

## Conflict of interest

The authors declare that they have no conflicts of interest with the contents of this article.
