# Supplementary material for: Triterpenoid derivatives inhibit Gli-mediated transcription in human glioblastoma cell line via direct interaction with Gli1
Source: J Biol Chem. 2025 Jul 10;301(9):110472. doi: 10.1016/j.jbc.2025.110472 (PMC12423687; doi:10.1016/j.jbc.2025.110472)
Supplement: Supporting information [file mmc1.docx]

Lupane derivatives inhibit Gli-mediated transcription in human glioblastoma cell line *via* direct interaction with Gli1

Ivo Frydrych ^1^, Jiří Řehulka ^1^, Lucie Borková ^2^, Jiří Hodoň ^1,2^, Jan Pokorný ^1, 2^, Veronika Šidová ^2^, Martina Medvedíková ^1^, Hanuš Slavík ^1^, Pavel Polishchuk ^1^, Tomáš Oždian ^1^, Jana Vrbková ^1^, Jan Šarek ^1^, Petr Džubák ^1,3^, Marián Hajdúch ^1,3^*, and Milan Urban ^1,2^*

^1^ Institute of Molecular and Translational Medicine, Faculty of Medicine and Dentistry, Palacky University and University Hospital Olomouc, Czech Republic

^2^ Department of Organic Chemistry, Faculty of Science, Palacky University Olomouc, Czech Republic

^3^ Institute of Molecular and Translational Medicine, Czech Advanced Technology and Research Institute, Palacky University Olomouc, Czech Republic

***** Correspondence: [milan.urban@upol.cz](mailto:milan.urban@upol.cz); [marian.hajduch@upol.cz](mailto:marian.hajduch@upol.cz)

**Supplementary Figures**

This supplementary file provides additional experimental details and supporting figures related to the data presented in the main manuscript.


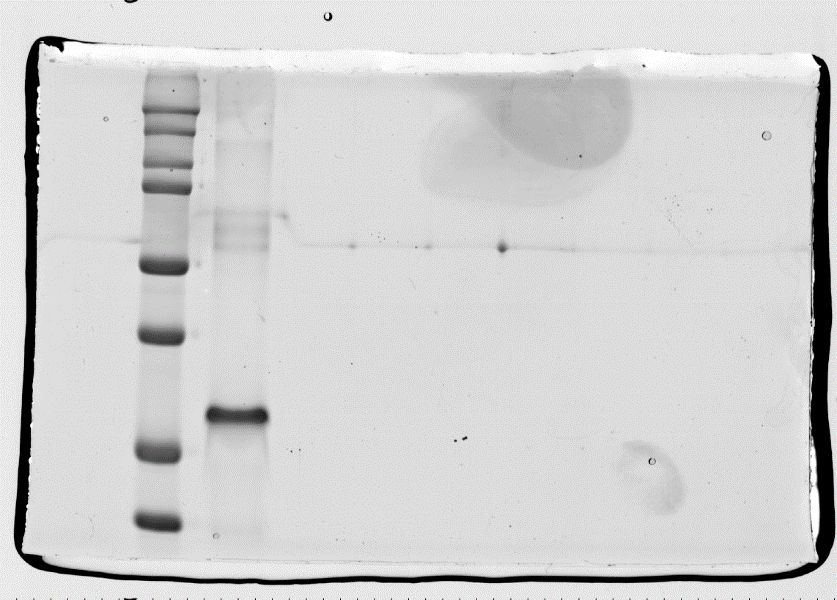


**kDa**

37

25

**Figure S1**. **SDS-PAGE analysis of recombinant Gli1 protein fragment (amino acids 222–400).**

The band corresponds to the purified Gli1 fragment used for ITC assays and confirms its apparent molecular weight and high purity. For details, see Supplementary Section S1.


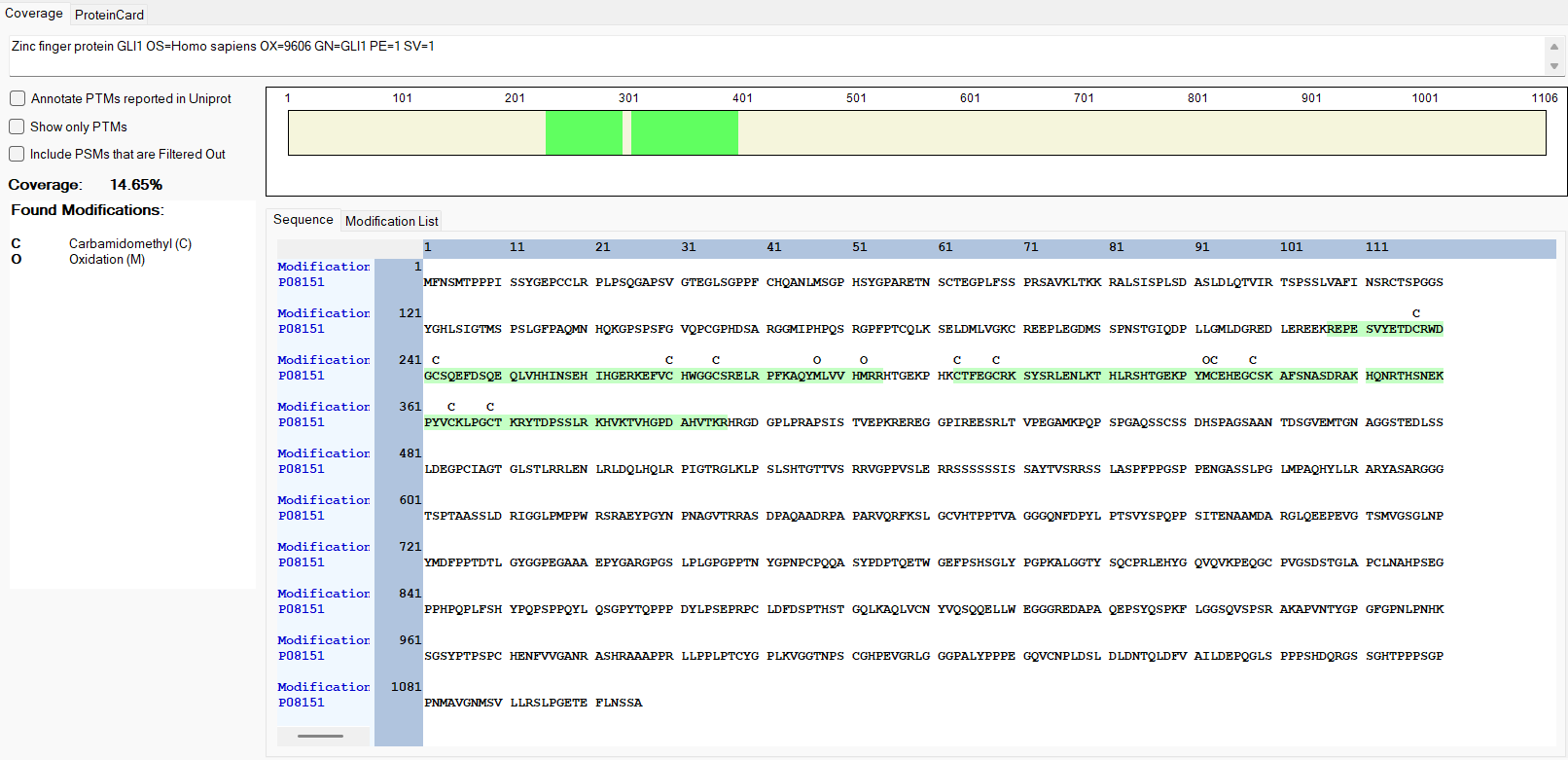


**Figure S2. Sequence coverage map of human Gli1 (UniProt P08151) identified by LC-MS/MS analysis of the recombinant fragment.**

The peptides identified cover the region corresponding to amino acids 227–396 of the protein fragment MBS2889276, confirming the identity of the recombinant construct. Overall sequence coverage of the recombinant fragment reached 88%. The full-length Gli1 protein is shown for reference.


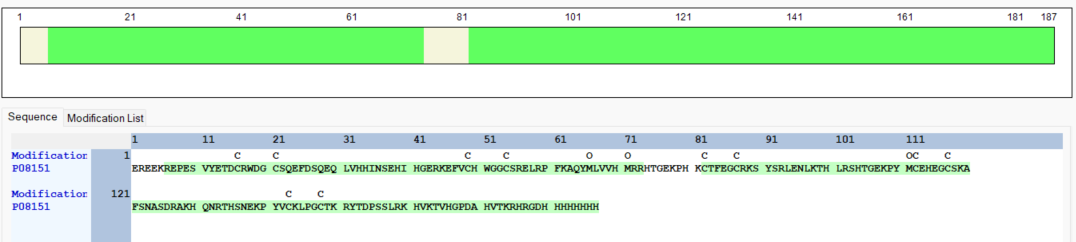


**Figure S3a. Sequence coverage of Gli1 with 8×His tag on the C-terminus.**

LC-MS/MS analysis confirms that the recombinant Gli1 fragment (residues 222–400) contains an 8×His tag on its C-terminus. The identified peptides (shown in green) cover 88% of the fragment’s sequence, confirming the identity and tag location of the expressed protein construct.


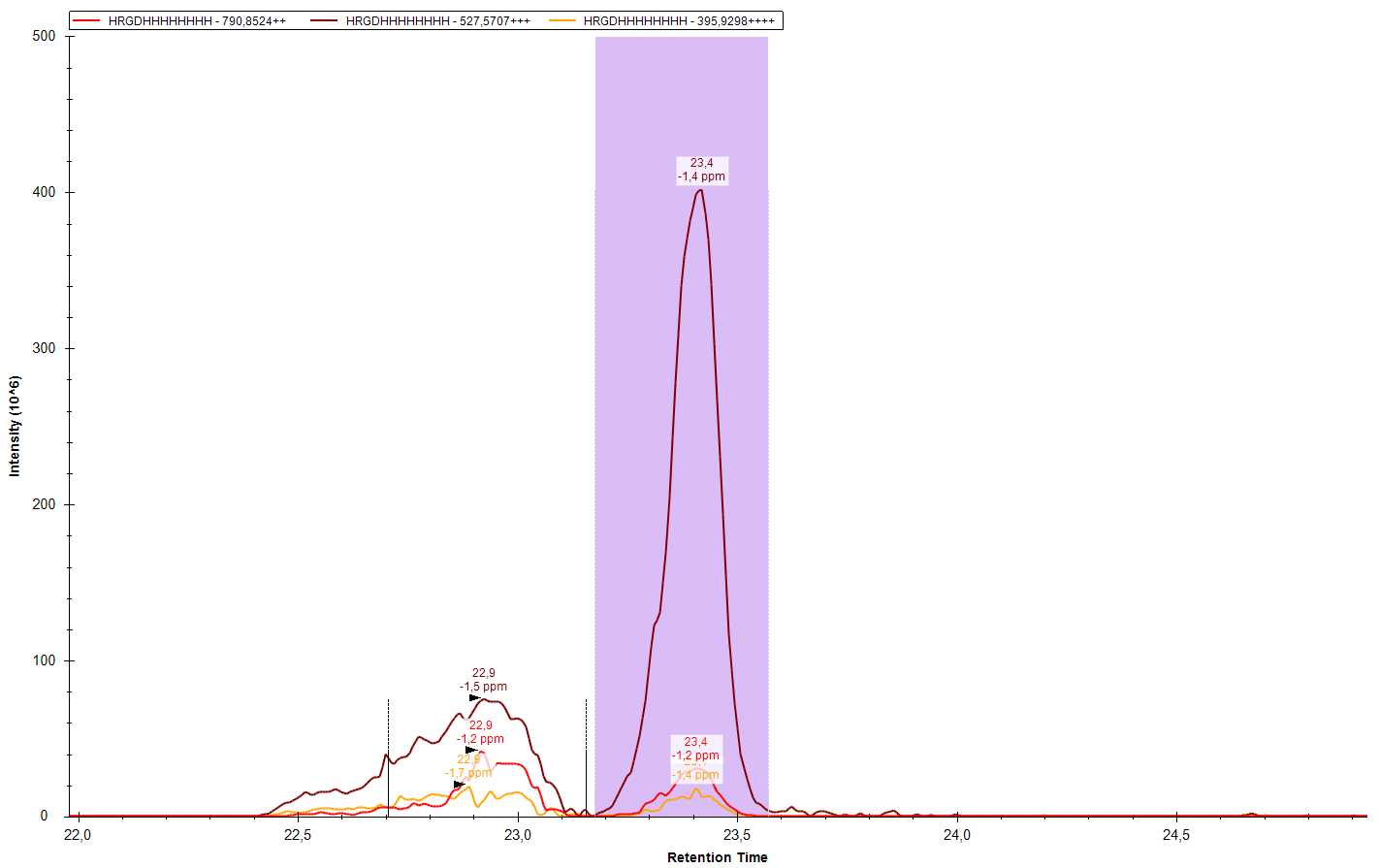


**Figure S3b. Skyline extracted peak area of the C-terminal 8×His-tagged peptide.**

Skyline analysis of the LC-MS/MS data reveals a robust peak corresponding to the His-tag peptide, demonstrating successful detection of the 8×His tag on the C-terminus of the recombinant Gli1 fragment.


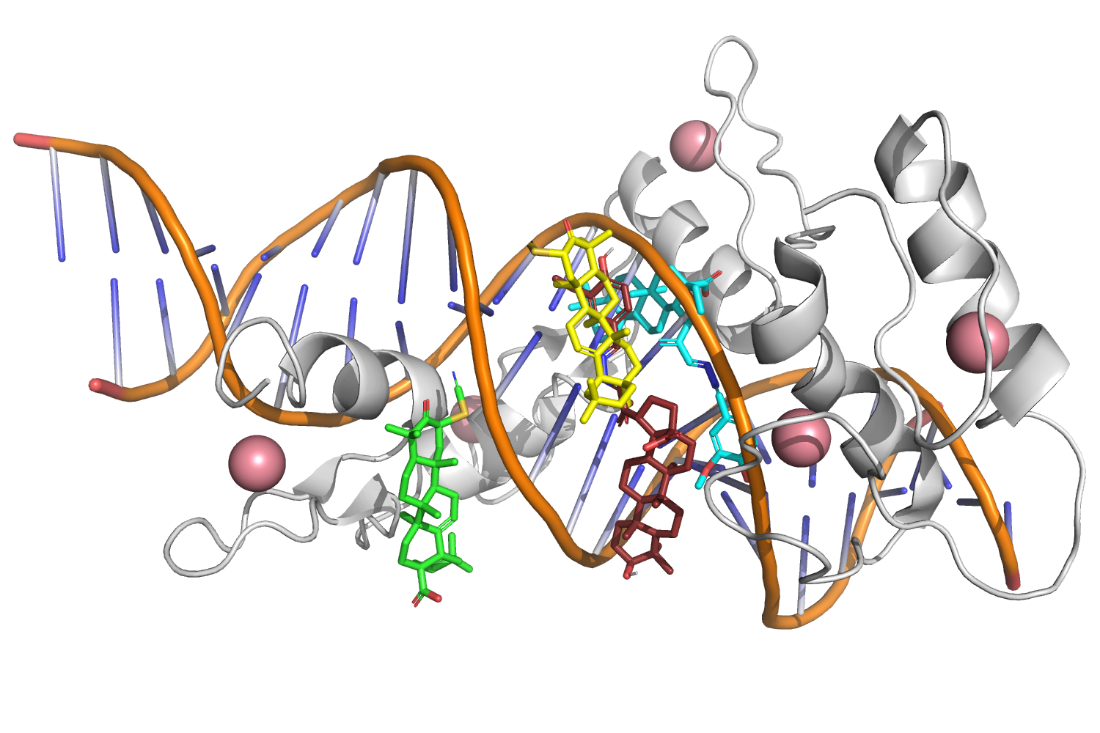


| Compound: | Docking into: | Color |
| --- | --- | --- |
| **15** | protein-DNA complex | yellow |
| **15** | Protein | green |
| **38** | protein-DNA complex | brown |
| **38** | Protein | cyan |

**Figure S4a. Color-coded summary of docking poses.**

Docking results are summarized for compounds 15 and 38 in the presence and absence of DNA. Colors indicate individual binding poses used for subsequent MD simulations.


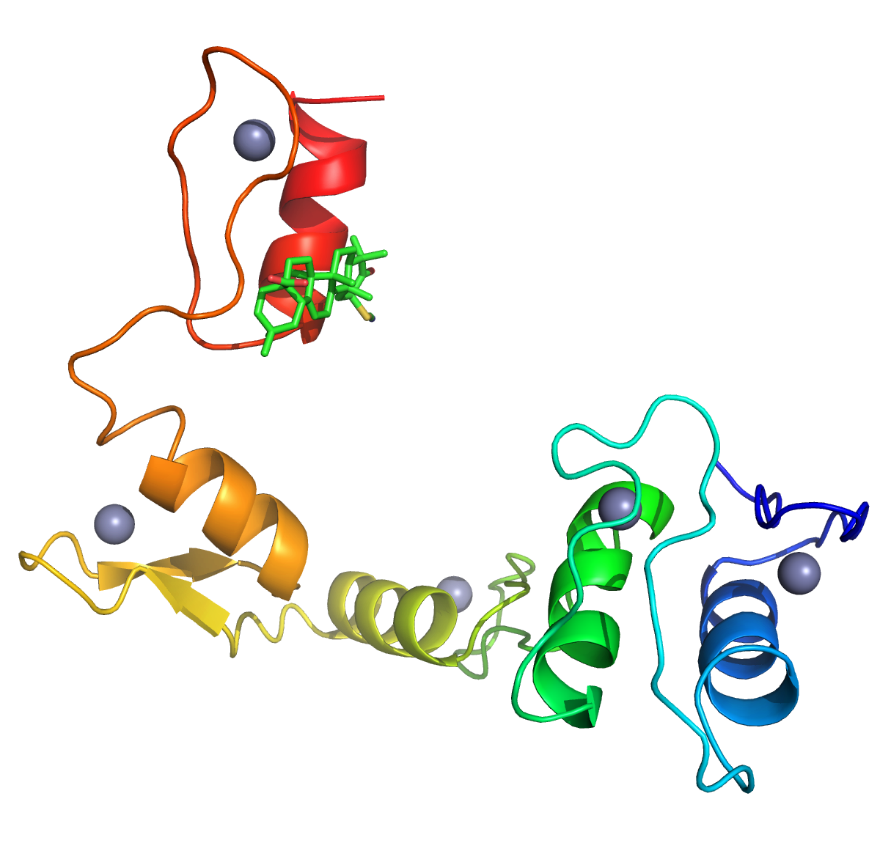


**Figure S4b. Representative start frame from MD simulation.**

Snapshot of the Gli1 protein (without DNA) showing the initial docking pose of compound 15 prior to the 50 ns MD simulation.


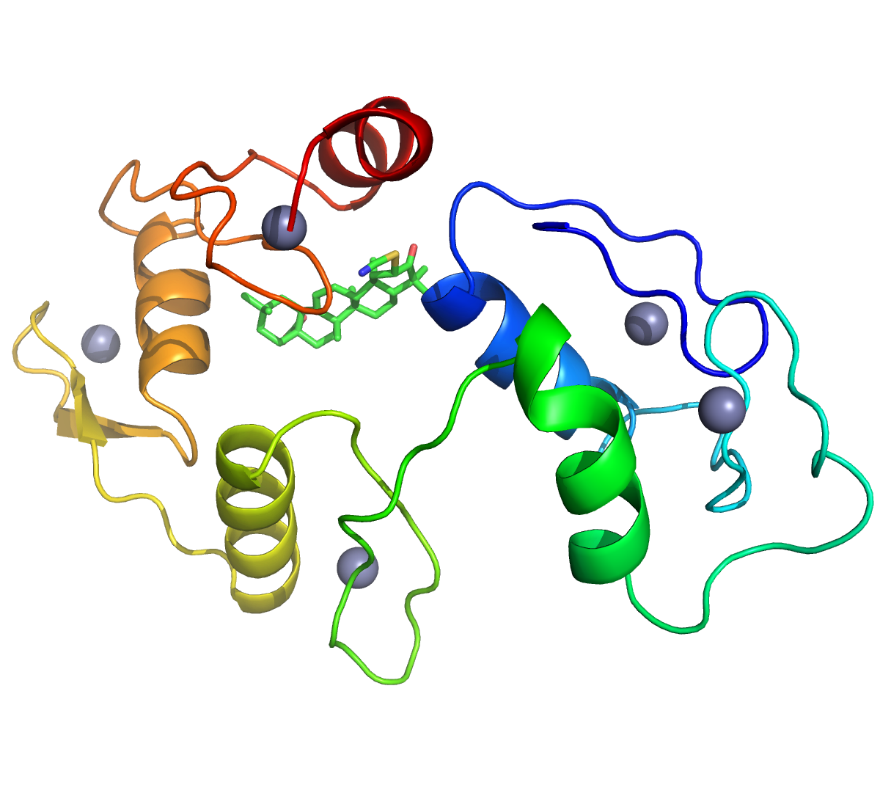


**Figure S4c. Representative end frame from MD simulation at 50 ns.**

Snapshot of the Gli1 protein (without DNA) showing the conformational state after 50 ns of MD simulation with compound 15.

**1 2 3 4 5 6 7 8 9 10**

**
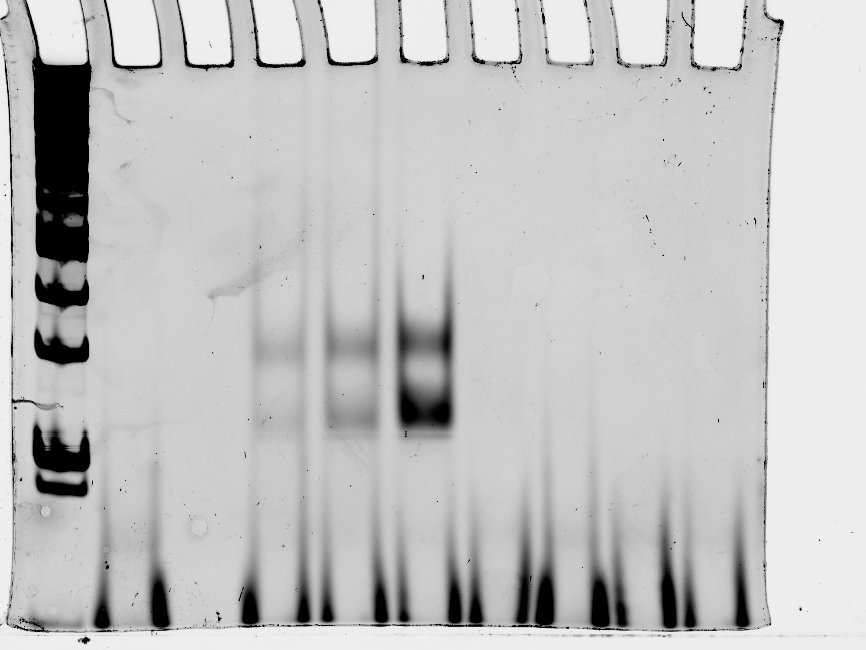
**

**Figure S5. Electrophoretic mobility shift assay (EMSA) demonstrating sequence-specific DNA binding of recombinant Gli1**

Lane assignment: 1 – DNA size ladder(ThermoFisher 1 kb Plus Ladder, 100–15,000 bp); 2 – Specific duplex DNA (0.5 µM); 3 – Gli1 protein only; 4–6 – Specific duplex (0.5 µM) + Gli1 (1.25, 2.5, 5 µM); 7 – Mutated duplex (0.5 µM); 8–10 – Mutated duplex (0.5 µM) + Gli1 (1.25, 2.5, 5 µM). All samples containing DNA (lanes 2 and 4–10) included the duplex at a fixed concentration of 0.5 µM. A concentration-dependent retardation of the electrophoretic mobility was observed for the GLI-binding site–containing duplex (lanes 4–6), indicating formation of specific DNA–protein complexes with the recombinant Gli1 zinc finger domain. In contrast, no mobility shift was detected for the mutant duplex (lanes 7–10), even at the highest protein concentrations, demonstrating the sequence specificity of the interaction. The DNA ladder in lane 1 includes fragments from 100 bp to 15 kb, with bands below 1 kb spaced at 100 bp intervals. Due to native PAGE conditions, the migration of DNA fragments deviates from standard agarose-based separation. DNA fragment sizes in this gel therefore serve as approximate references only.

**1 2 3 4 5 6 7 8 9 10**

**
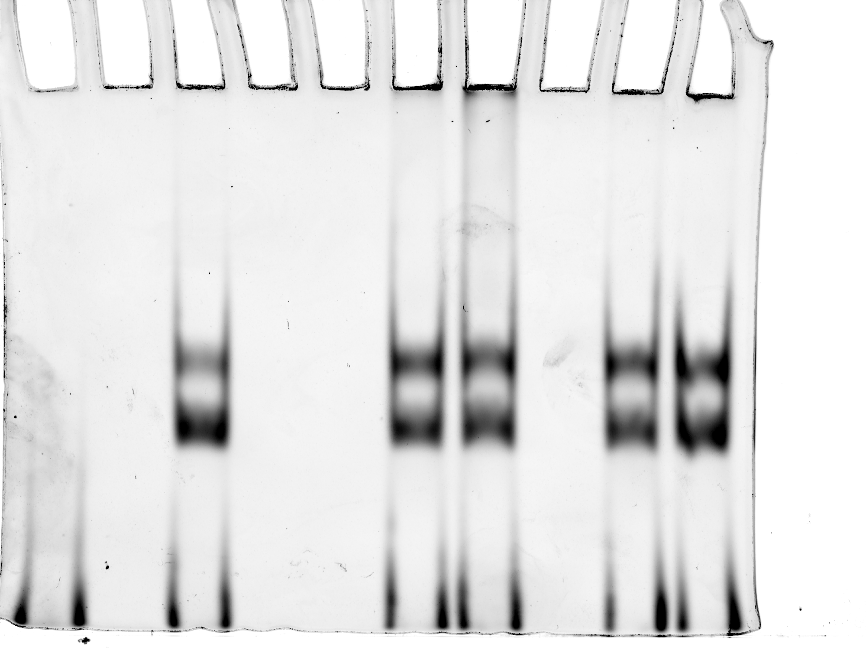
**

**Figure S6. EMSA analysis of the effect of compounds 15 and 38 on Gli1–DNA complex formation.**

Lane assignment: 1 – Specific duplex DNA (0.5 µM); 2 – Gli1 protein only; 3 – Duplex + Gli1 (5 µM); 4–5 – Empty; 6 – Duplex + Gli1 + 10 µM compound **15**; 7 – Duplex + Gli1 + 50 µM compound **15**; 8 – Empty; 9 – Duplex + Gli1 + 10 µM compound **38**; 10 – Duplex + Gli1 + 50 µM compound **38**. The addition of compounds **15** or **38** at the indicated concentrations did not alter the electrophoretic mobility of the DNA–Gli1 complex, suggesting that DNA binding was not impaired under these conditions.

**Supplementary Experimental Procedures and Results**

**S1. Verification of Gli1 fragment size and purity by SDS-PAGE**

The recombinant Gli1 protein used for ITC experiments (MyBioSource, Cat. No. MBS2889276) corresponds to a fragment encompassing amino acids 222–400 of human Gli1 (UniProt P08151). SDS-PAGE was performed on a 10% polyacrylamide gel under denaturing conditions and visualized by Coomassie Brilliant Blue staining. This analysis confirmed the protein’s high purity (>90%) and its apparent molecular weight. Although the calculated size of the fragment is approximately 20.8 kDa, the band consistently migrated at ~30 kDa, which is in agreement with the reference data provided by the supplier. This discrepancy is likely caused by the protein’s sequence composition and partial intrinsic disorder. For gel documentation, see Figure S1.

**S2. MS identification of the recombinant Gli1 protein fragment**

Method:

An aliquot of the Gli1 fragment stock solution (MyBioSource, Cat. No. MBS2889276) was digested *in-solution* using a trypsin/Lys-C mix. The resulting peptides were analyzed by nanoHPLC (Ultimate 3000 – Thermo Fisher Scientific) coupled to a Thermo EasySpray 75 µm × 25 cm column and a Orbitrap Fusion mass spectrometer (Thermo Fisher Scientific) operated in DDA mode. Data were processed using Proteome Discoverer 3.2 (Thermo Fisher Scientific).

Results:

A total of 32 unique peptides were identified, mapping to the human Gli1 protein (UniProt P08151), with an overall sequence coverage of 15% when compared to the full-length protein (Figure S2). Importantly, the identified peptides covered the region corresponding to residues 227–396 of the recombinant fragment, representing 88% sequence coverage of the studied construct. Only the N- and C-terminal regions and a small internal segment lacked detected peptides. The missing internal peptides (HTGEK and PHK) are likely too small for reliable detection under the applied proteomics search criteria. The N- and C-terminal peptides may also contain modifications that were not captured in the search, possibly including partial His-tag sequences.

**S3. Confirmation of the C-terminal His-tag on the recombinant Gli1 protein**

Method:

The presence of an 8×His tag on the C-terminus of the recombinant Gli1 fragment was confirmed using targeted proteomics. Data from the LC-MS/MS analysis (described in S2) were further analyzed with Skyline software, applying a variable search for histidine-rich peptides at both termini. The resulting targeted peptide was then confirmed using Thermo Proteome Discoverer 3.2 with a custom FASTA sequence including the expected His-tag.

Results:

The targeted search identified the expected His-tag peptide on the C-terminus of the recombinant Gli1 fragment, confirming the presence of the 8×His tag at this location. Sequence coverage of the tagged protein construct was further visualized, and extracted peak areas of the His-tag peptide were consistent with the recombinant design (see Figure S3).

**S4. Molecular docking and MD simulations of Gli1–ligand complexes**

Method:

Molecular docking of compounds 15 and 38 to the Gli1 zinc finger domain (PDB ID: 2GLI) was performed using the GNINA program. Docking was conducted with the DNA motif present and absent to explore possible binding modes. Color codes for docking poses are summarized in Figure S4a. The top 10 docking poses were subjected to molecular dynamics (MD) simulations using GROMACS (50 ns, three replicates). Snapshots from the start and the end of the MD simulations are shown in Figures S4b and S4c, respectively.

Results:

Docking suggested that compounds 15 and 38 interact with shallow pockets near the DNA-binding domain of Gli1, consistent with potential interference in DNA binding. MD simulations showed significant conformational flexibility of the protein, with some ligand poses remaining stable and others dissociating during the trajectory. These findings are consistent with the partial resolution and inherent flexibility of the Gli1 protein and align with the discussion presented in the main text.

**S5. Electrophoretic mobility shift assay (EMSA) confirming DNA binding of recombinant Gli1**

Method:

EMSA was performed to validate the biological activity and DNA-binding capacity of the recombinant Gli1 zinc finger domain (residues 222–400) used in biophysical assays. A 27-mer duplex oligonucleotide containing a previously validated GLI-binding site (adapted from Maresca et al., 2023; *Pharmacol Res*, 195:106858) and mutated control sequence were synthesized and annealed. The sequences used were as follows:

- **GLI1-specific forward**: 5'-AAGTAGAGAGACCACCCAGGTAGGCAA-3'
- **GLI1-specific reverse**: 5'-TTGCCTACCTGGGTGGTCTCTCTACTT-3'
- **GLI1-mutated forward**: 5'-AAGTAGAGAGACTATCCAGGTAGGCAA-3'
- **GLI1-mutated reverse**: 5'-TTGCCTACCTGGATAGTCTCTCTACTT-3'

The mutated duplex was designed in-house and carries base substitutions within the consensus GLI-binding region to disrupt recognition. Binding reactions (20 µl) were assembled in optimized binding buffer and incubated for 25 minutes at room temperature with increasing concentrations of Gli1 (1.25–5 µM). Complexes were resolved by electrophoresis on a 6% native polyacrylamide gel in TBE buffer (1x), run at 120 V for 60 minutes at 4 °C. Following electrophoresis, gels were stained with SYBR Green (1:10,000 dilution in TBE) for 20 minutes and imaged using a Typhoon FLA 9000 fluorescence scanner.

Results:

Recombinant Gli1 (residues 222–400) was evaluated for its DNA-binding capacity using EMSA with a synthetic 27-mer duplex containing a validated GLI-binding motif. A progressive shift in DNA mobility was observed upon incubation with increasing Gli1 concentrations, consistent with specific complex formation. No shift was detected with a mutant duplex, confirming sequence specificity. These results validate the functional activity of the recombinant protein used in ITC experiments. Attempts to visualize protein–DNA complexes using SYPRO Ruby staining were not successful, likely due to the small size (~20 kDa) and native conformation of the Gli1 zinc finger fragment. This limitation is well recognized for native PAGE systems, especially when analyzing low-molecular weight proteins. Since EMSA primarily relies on shifts in DNA migration to infer complex formation, DNA staining with SYBR Green was used as the primary readout of specific Gli1–DNA interactions.

**S6. Compounds 15 and 38 do not interfere with Gli1–DNA complex formation in EMSA.**

Method:

To evaluate whether compounds **15** and **38** interfere with the DNA-binding activity of Gli1, EMSA was performed using a 27-mer DNA duplex containing a validated GLI-binding motif. Recombinant Gli1 protein (residues 222–400, 5 µM) was preincubated with either compound **15** or **38** at 10 or 50 µM in binding buffer for 20 minutes at room temperature. Subsequently, the DNA duplex (0.5 µM final concentration) was added, and the reaction was incubated for an additional 25 minutes. Binding reactions (20 µl) were then resolved on a 6% native polyacrylamide gel (1× TBE) at 120 V for 60 minutes at 4 °C. DNA bands were visualized by post-staining with SYBR Green and imaged using a Typhoon FLA 9000 fluorescence scanner.

Results:

No change in electrophoretic mobility was observed in samples containing Gli1 and either compound **15** or **38**, compared to the positive control (Gli1 + DNA without inhibitor). These results indicate that neither compound disrupted the formation of DNA–Gli1 complexes under the conditions tested, suggesting that their mechanism of action may differ from that of classical DNA-binding antagonists.
